# Harnessing miRNA therapeutics: a novel approach to combat heart and brain infarctions in atherosclerosis

**DOI:** 10.1038/s41420-025-02649-9

**Published:** 2025-10-24

**Authors:** Jie Wang, Yinghui Li, Haoxuan Wang, Qian Meng, Peiyu Li, YuQin Wang, Kun Wang, SuMin Yang

**Affiliations:** Department of Cardiovascular Surgery, Institute of Chronic Diseases, The Affiliated Hospital of Qingdao University, College of Medicine, Qingdao University, Qingdao, China

**Keywords:** Atherosclerosis, miRNAs

## Abstract

MicroRNAs (miRNAs) are small non-coding RNAs that regulate gene expression and play critical roles in various cellular processes. Increasing evidence suggests that miRNAs are involved in the development and progression of atherosclerosis, which is the leading cause of myocardial infarction and stroke. These molecules influence key pathological mechanisms, including lipid metabolism, endothelial dysfunction, vascular inflammation, and plaque stability. This review summarizes the role of miRNAs in atherosclerosis-induced cardiac and cerebral infarction and explores their potential as therapeutic targets. We discuss emerging miRNA-based interventions, such as miRNA mimics and inhibitors, which offer promising strategies for disease prevention and treatment. Understanding the regulatory functions of miRNAs in cardiovascular and cerebrovascular events may provide new insights for developing innovative therapies aimed at reducing the burden of atherosclerosis-related diseases.

## Facts

- miRNA affects atherosclerosis by influencing endothelial cell aging, foam cell formation, vascular smooth muscle cell proliferation and migration.
- miRNA affects the development process of myocardial infarction by influencing the repair of myocardial cells, the generation of new blood vessels, and inflammatory responses.
- miRNA affects cerebral infarction by regulating neuronal apoptosis, glial cells and their inflammatory response, blood-brain barrier and neovascularization recovery.
- The miRNA in different cells can be up-regulated or down-regulated by means of chemotherapy, exosome targeted delivery and other means, so as to improve the myocardial infarction and cerebral infarction caused by atherosclerosis.

## Introduction

MicroRNAs, also known as miRNAs, were first discovered in 1993 in the nematode *Caenorhabditis elegans*. They are small non-coding RNAs with a length of approximately 22 nucleotides. MiRNAs originate in the nucleus, where RNA polymerase II transcribes primary miRNA transcripts (pri-miRNAs). Subsequently, they are cleaved into precursor miRNAs by microprocessors and transferred to the cytoplasm for further processing into single-stranded RNAs, known as mature miRNAs. A single miRNA can modulate the pathways of the entire cell by interacting with a wide range of target genes [1]. Although other mechanisms of action have been proposed, most miRNAs function by binding to the 3′ untranslated region (3′ UTR) of the target messenger RNA (mRNA), which is subsequently degraded or translationally repressed by the RNA-induced silencing complex (RISC) [2]. Rather than completely silencing their target genes, this binding typically results in modest downregulation. Since the discovery of miRNAs, their regulatory roles in various diseases have been extensively reported, including cancer [3], immunotherapy [4], glomerulonephritis [5], schizophrenia [6], and diabetes [7], among others. Research has shown that miRNAs can target genes involved in vascular remodeling processes, such as cell proliferation, apoptosis, motility and the production or degradation of the extracellular matrix. These regulatory effects are categorized as either physiological or pathological, encompassing beneficial adaptive responses to changes in hemodynamics, vasoactive substances, or cytokines, as well as maladaptive responses that may contribute to cardiovascular disease (CVD) [8]. Thus, miRNAs have been mechanistically linked to play a critical regulatory role in the development of atherosclerosis (AS) and post-ischemic neovascularization.

Atherosclerotic disease is the leading global cause of mortality and the common pathological basis for major vascular disorders, including myocardial infarction and cerebral infarction. Its hallmark features include lipid deposition, immune cell infiltration, and sustained inflammatory responses. Numerous risk factors contribute to atherosclerosis, such as hypertension, dyslipidemia, and insulin resistance. Current consensus posits that these factors interact with arterial wall cells to drive chronic vascular inflammation, with endothelial cells, macrophages (a subset of leukocytes), and vascular smooth muscle cells (VSMCs) constituting the principal cellular mediators of disease progression. The pathogenesis of atherosclerosis involves multifaceted mechanisms. Early lesions emerge beneath an intact endothelial layer, where low-density lipoprotein (LDL) particles traverse dysfunctional endothelial cells into the subendothelial space. Following extravasation, these particles undergo retention and biochemical modification (e.g., oxidation), triggering pro-inflammatory signaling cascades [9]. Subsequently, upregulated expression of adhesion factors (notably VCAM-1) in endothelial cells promotes monocyte recruitment to the arterial wall, representing one of the earliest events in atherogenesis. Within the subendothelial space, monocytes differentiate into macrophages. These macrophages amplify inflammation by secreting monocyte chemoattractant protein-1 (MCP-1), a potent chemokine that drives further leukocyte infiltration. In the intima of blood vessels, mature macrophages transform into foam cells rich in LDL particles through phagocytosis, which is a sign of early atherosclerosis. Foam cells further accumulate lipids, and eventually release cholesterol through apoptosis or necrosis, or transport cholesterol through membrane transporters, leading to lipid accumulation in plaque [10]. In the intermediate and advanced stages, smooth muscle cells with repair and protective abilities enter the intima and synthesize collagen-rich extracellular matrix components, while proliferating and aggregating in the arterial wall. This excessive repair response strengthens arterial plaque stability to some extent, but it also results in the luminal stenosis and a reduction in blood flow. Smooth muscle cells also transform to other cell types (called phenotypic modulation), exhibiting a variety of cancer-like hallmarks, such as hyperproliferation [11]. As cholesterol deposits and cellular debris progressively accumulate within the intima, atherosclerotic plaques undergo expansion. A subset of these plaques develop structural instability, predisposing them to rupture—an event that triggers vascular occlusion and thrombus formation, constituting the primary driver of mortality in atherosclerosis (AS). The pathogenic cascade originates from endothelial injury, initiating lipid deposition and oxidative modification within the subendothelial space. These pathological changes recruit circulating monocytes (which differentiate into macrophages) and induce vascular smooth muscle cell migration toward the lesion site, ultimately culminating in the formation of complex atherosclerotic plaques [12].

Accumulating evidence has established a robust association between miRNAs and atherosclerotic pathogenesis. miRNAs exert multifaceted regulatory effects on atherogenesis through distinct mechanisms, including modulating endothelial dysfunction [13], attenuating macrophage-mediated intracellular cholesterol accumulation [14], and controlling VSMC proliferation and migratory dynamics [15]. Based on plaque stability, it can be categorized into stable plaques and vulnerable plaques. Vulnerable plaques are a class of plaques that are prone to rupture and ruptured. Once affected by emotions, exercise, temperature, etc., the hemodynamic stressors or the blood flow will impact on the vessel wall, as a result, the lipids and other substances within the plaque will gush out, forming a thrombus that will block the blood vessel. If the thrombus blocks the coronary artery, it will cause acute myocardial infarction; if it blocks the cerebral blood vessels, it will cause cerebral infarction. Stable plaque surface is thicker and not easy to rupture, but if the stable plaque gradually increases, it will also lead to the gradual narrowing of the lumen of the blood vessel, or even completely block the blood vessel, leading to the interruption of blood supply, causing the occurrence of heart infarction, cerebral infarction, and other diseases [16]. This suggests that miRNAs also play different roles in the regulation of heart infarction and cerebral infarction triggered by atherosclerosis.

This review delineates the pivotal regulatory roles of miRNAs in atherosclerosis and its downstream complications, including myocardial infarction and ischemic stroke. miRNAs exert dual regulatory functions throughout disease progression by orchestrating gene expression networks that govern vascular endothelial homeostasis, inflammatory cascades, and plaque stability. Specific miRNA families demonstrate protective effects through suppression of oxidative stress and pro-inflammatory signaling (e.g., NF-κB), whereas others exacerbate atherosclerotic lesion progression by promoting macrophage lipid retention or fibrous cap thinning. Emerging therapeutic strategies leveraging miRNA mimics or inhibitors (e.g., antagomirs) represent novel paradigms for precision modulation of atherogenesis. Furthermore, the tissue specificity and circulatory stability of miRNAs position them as promising diagnostic biomarkers for subclinical atherosclerosis. Collectively, this synthesis advances translational insights into miRNA-based interventions for cardiovascular and cerebrovascular events, while establishing a conceptual framework for developing next-generation therapeutics targeting epigenetic regulatory axes.

## miRNA causes Heart and Brain Infarction through Atherosclerosis

**Fig. 1:**
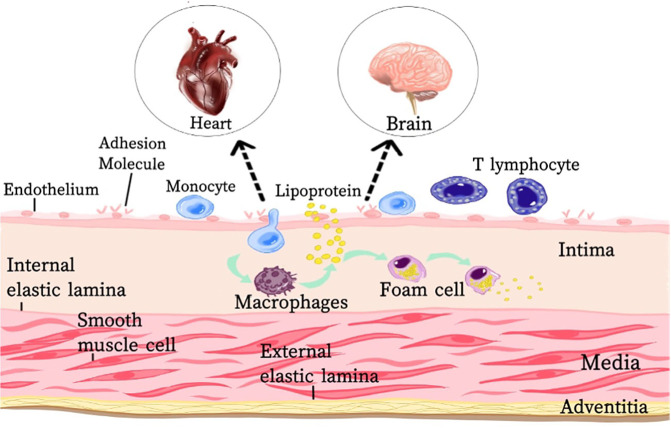
miRNA causes heart and brain infarction through atherosclerosis. This figure shows the development process of atherosclerosis and the main participating cells (Endothelial cells damage, vascular smooth muscle cells proliferation and migration, macrophages recruitment). Eventually, plaques are formed in cardiovascular and cerebral vessels, which in turn induce myocardial infarction and cerebral infarction. MiRNAs regulate atherosclerosis by affecting these cells.

According to recent studies, miRNAs can regulate gene expression changes observed during atherosclerosis, and these miRNAs can be used as biomarkers for early detection of atherosclerosis and targeted therapy. The pathophysiology of AS is modulated by multiple miRNAs, which dichotomously regulate a wide range of biological processes including initiation (endothelial senescence and inflammation), progression (SMC proliferation and migration, increased necrosis of lipid nuclei within plaques, angiogenesis), and endpoint (unstable plaque rupture). In short, with the involvement of miRNAs, endothelial cell function, macrophage recruitment and transformation, and VSMC activity are affected, which in turn promotes or inhibits plaque formation, enlargement, or even rupture, and the formation of thrombus, triggering AMI or cerebral infarction (Fig. 1).

### miRNAs and endothelial cells

Early atherosclerotic lesions originate from endothelial dysfunction. Emerging research demonstrates that multiple miRNAs regulate endothelial senescence in AS through distinct molecular mechanisms, thereby driving inflammatory cascades (Table 1). Certain miRNAs exhibit pro-atherogenic effects by accelerating endothelial cell senescence and apoptosis, either through repression of anti-aging genes or activation of pro-inflammatory pathways. Among them miR-132/212 promotes AS progression via PTEN suppression, amplifying platelet-derived growth factor BB (PDGF-BB)-induced VSMC proliferation and migration. miR-503-5p, transported via macrophage-derived extracellular vesicles (EVs), suppresses human coronary artery endothelial cell (HCAEC) proliferation/angiogenesis while enhancing human coronary artery smooth muscle cell (HCASMC) migratory capacity. MiR-126-5p in endothelial EVs mediates radiation-induced inflammatory crosstalk to monocytes, increasing monocyte-endothelial adhesion and pro-atherogenic macrophage differentiation. MiR-23a-3p synergistically activates NF-κB and p38/MAPK pathways through TNFAIP3 targeting, concurrently stimulating endothelial apoptosis and inflammasome signaling. This suggests that one miRNA can affect one or even more processes in AS. Some miRNAs, on the other hand, can inhibit the development of atherosclerosis by targeting and repressing genes involved in endothelial cell apoptosis or by suppressing the inflammatory response. Among them, miR-146a-encapsulated liposomes attenuate vascular inflammation in human aortic endothelial cells (HAECs) and VSMCs via ICAM-1 downregulation and monocyte adhesion inhibition. miR-26 exerts dual anti-atherogenic effects by targeting COL1A1, HMGA1/B1, and MALT1 to suppress NF-κB activity, thereby reducing IL-1β, IL-6, and TNF-α production; inhibiting endothelial proliferation and VSMC differentiation through SMAD1/SMAD4 repression, ultimately diminishing plaque burden.

**Table 1:** Regulation of atherosclerosis by miRNAs.

miRNA	target gene	Signaling pathway	Corresponds	Reference
miR-125a-5p	RTEF-1	Pink1-Mfn2-Parkin access road	Inhibition of mitochondrial autophagy	[88]
miR-132/212	Gna12; PTEN	GNA12/PTEN signaling	Exacerbates EC apoptosis; promotes VSMC proliferation and migration	[89]
miR-503-5p	Smad7	Smad7-Smurf1/Smurf2-TGF-β axis	Reduced EC proliferation and angiogenesis and promoted SMC proliferation and migration.	[90]
miRNA-130a	PPARγ	PPARγ/NF-κB	promote inflammation	[91]
miR-23a-3p	TNFAIP3	NF-κB and p38/MAPK	Promotes inflammation and endothelial cell apoptosis	[92]
miR-92a	SIRT1 and KLF2		Promotes endothelial cell senescence	[93]
miR-217	APLNR and VEGFR1	eNOS Activator Network	exacerbate atherosclerosis	[94]
miR-126-5p			promote inflammation	[95]
miR-652-3p	Ccnd2		Inhibition of EC proliferation	[96]
miRNA let-7b	HAS-2	P13K/Akt access	Inhibition of EC apoptosis	[97]
miR-181a-5p/3p	TAB2 and NEMO	NF-κB	Reducing inflammation	[98]
miR-146a	ICAM-1		Reducing inflammation	[99]
miR-26	SMAD1 and SMAD4, etc.		Inhibition of EC growth, angiogenesis and VSMC differentiation	[100]
miR-21-5p	SKP2	SKP2/EP300/ HMGB1	Enhances macrophage polarization and promotes inflammation	[101]
miR-30b-5p	UBE2D2	UBE2D2/KAT2B/ HMGB1	Promote polarization of pro-inflammatory cells and recruitment of macrophages	[102]
miR-30a-3p	ABCA1		Promote foam cell formation	[103]
miR-216a	Smad3	NF-κB	Facilitated conversion of M2 to M1	[104]
miR-127-3p	SCD-1	SCD1/UFA	Promote macrophage proliferation	[105]
miR-520a-3p	UVRAG	IL4/IL13	Reduction of macrophage autophagy	[106]
miR-146a	IGF2BP1 and HuR		Reduced macrophage migration	[107]
miR-369-3p	GPR91	Succinate-GPR91-HIF-1α-inflammasome signaling axis	Reduces mitochondrial damage and inflammation	[108]
miR-21	MKK3	p38-CHOP and JNK signaling	Enhancement of macrophage apoptosis	[109]
miR-155-5p	CD36 and Vav3; SOCS1	KEGG pathway	Inhibition of ox-LDL uptake; increased macrophage cholesterol efflux	[110]
miR-382-5p	BMP4	PPARγ-ABCA1 /ABCG1	Foam cell formation decreases and cholesterol outflow increases	[111]
miR-1914-5p	ICAM, Mac-1, MCP-1		Inhibition of monocyte recruitment	[112]
miR-140-5p	ROBO4	ROBO4/VEGF signaling pathway	Inhibition of VSMC apoptosis	[17]
miR-214	Smad7	TGF-β/Smad pathway	Promotes VSMC proliferation, migration, contraction, hypertrophy and stiffness	[18]
miR-92a	KLF4		Inhibition of VSMC proliferation and migration	[19]
miR-17	IRF9		ditto	[113]
miR-146b	Bag1 and Mmp16		ditto	[114]
miR-214	NCKAP1		ditto	[115]
miR-663	JunB	JunB/myosin light chain 9	ditto	[116]
miR-223-3p	STAT3	IL-6/STAT3	Blocking osteogenic transformation and calcification in VSMC	[117]
miR-99a-5p	HOXA1		Inhibition of ASMC proliferation, migration and invasion	[118]
miR-15a-5p /199a-3p	IKKα, IKKβ and p65	NF-κB	Reduced ox-LDL uptake and inflammation in VSMC.	[20]
Let-7d-5p	OLR	NF-κB	Reduced ox-LDL uptake in VSMC and maintained the contractile phenotype	[21]

### miRNAs and macrophages

Atherosclerosis arises from chronic inflammatory processes characterized by macrophage maturation, LDL uptake, and subsequent foam cell formation. The accumulation of cholesterol-laden foam cells within the intima of large arteries marks the onset of early “fatty streak” lesions, which progressively recruit additional cell types and evolve into complex multicellular atherosclerotic plaques. Specific miRNAs exacerbate inflammation and foam cell pathology through distinct mechanisms (Table 1). Certain miRNAs drive macrophage polarization and recruitment via post-translational modifications (e.g., HMGB1 acetylation) mediated by target gene networks, while others impair cholesterol efflux through transcriptional repression of ATP-binding cassette transporters (e.g., ABCA1). Notably, some miRNAs amplify pro-inflammatory M1 macrophage polarization via NF-κB pathway activation, thereby accelerating plaque progression.

Conversely, protective miRNAs counterbalance these effects by attenuating inflammatory cascades and foam cell dynamics. Key mechanisms include: mitigating macrophage oxidative damage through redox-sensitive gene regulation; enhancing cholesterol efflux via ABCA1/ABCG1 upregulation; and suppressing monocyte-to-macrophage differentiation and recruitment. These regulatory axes collectively modulate the inflammatory microenvironment critical for atherosclerosis pathogenesis.

### miRNAs and vascular smooth muscle cells

One of the key events in atherosclerosis is the shift of SMC from a contractile phenotype to a synthetic phenotype. MiRNAs can exert atherosclerosis regulation by accelerating or inhibiting the phenotypic transition (Table 1). Some miRNAs promote SMC proliferation and migration and inhibit SMC apoptosis, thereby accelerating the disease progression. For example, high levels of miR-140-5p promotes VSMC proliferation, migration, and invasion, and inhibits VSMC apoptosis by reducing ROBO4 expression [17]; miR-214 inhibits the level of its target gene Smad7, thereby negatively regulating the TGF-β/Smad pathway. Meanwhile, miR-214 established crosstalk between angiotensin II (Ang II)-induced AT1R signaling and TGF-β-induced TGF-β/Smad signaling. Knocking out miR-214 can inhibit a series of changes induced by Ang II in VSMCs, such as proliferation, migration, swelling, and stiffness [18]. MiR-92a regulates VSMC to a synthetic phenoty through Kruppel-like factor 4 (KLF4) pathway and promotes VSMC proliferation and migration [19]. Some miRNAs can inhibit the transition of VSMC from a contractile to a synthetic phenotype, thereby slowing down the development of atherosclerosis by the mechanism of inhibiting the proliferation and migration of VSMCs, which mechanisms are through modulating of interferon regulatory factors, anti-apoptotic genes, matrix metalloproteinases, and differentiation marker genes. In addition, miR-15a-5p and miR-199a-3p reduced ox-LDL uptake and NF-κB-regulated inflammation in VSMC [20]. Overexpression of let-7d-5p in HAoSMC resulted in a decrease in the number of ox LDL receptor OLR1 on the cell membrane, thereby attenuating pro-inflammatory signaling cascades [21].

## miRNA and Myocardial Infarction

Atherosclerotic plaque occlusion or rupture represents the initial pathological event precipitating myocardial infarction. Subsequent thrombosis causes vascular obstruction, compromising systemic circulation and end-organ perfusion. Prolonged ischemia triggers rapid infiltration of neutrophils and inflammatory monocytes into the affected myocardium, resulting in hypoxia-induced cardiomyocyte apoptosis and necrosis. This cellular demise precipitates acute cardiac dysfunction, which may progress to maladaptive ventricular remodeling and ultimately chronic heart failure. Repair of cardiomyocytes, neovascularization, and inhibition of the inflammatory response in a timely manner are the three critical components of this process.

### Myocyte Repair

In the adult mammalian heart, the majority of cardiomyocytes exist in a terminally differentiated state with severely restricted proliferative potential, rendering the heart incapable of meaningful regeneration following ischemic injury. Recovery of cardiomyocyte numbers after myocardial infarction can be achieved by inhibiting cardiomyocyte apoptosis, promoting cardiomyocyte proliferation, and mediating progenitor cell differentiation and reprogramming of non-cardiomyocytes (e.g., fibroblasts). The number of miRNAs has been found to be involved in this process (Table 2).

**Table 2:** Regulation of infarction by miRNAs.

miRNA	Target gene	Signaling pathway	Corresponds	Reference
miR-103-3p	Hlf	Hlf/Fyco1	Inhibition of autophagy and promotion of apoptosis in cardiomyocytes	[119]
miR-503	PGC-1β and SIRT3		Promotes cardiomyocyte death	[120]
miRNA-542-5p	ATG7		Inhibition of autophagy thereby exacerbating cardiomyocyte damage	[121]
miR-24-3p	HO1	circCHSY1/miR-24-3p/ HO1	Aggravated mitochondrial damage in cardiomyocytes	[122]
miR-141-3p	CHD8	CHD8/p21	Attenuates cardiomyocyte apoptosis	[123]
miRNA-146a	NOX4	NOX4/P38	Reduction of oxidative stress damage in cardiomyocytes	[124]
miR-663b	BCL2L1		Reduction of cardiomyocyte apoptosis	[125]
miR-450b-5p	ACSL4		Reduction of cardiomyocyte iron death	[126]
miR-181a	HK2；Adamts1；PDCD4	Ngal/Aldo-MR; PDCD4/BID	dual role	[27–29]
miRNA-21	Ajuba	ajuba/Isl1 axis access road	Differentiation of BMSCs to cardiomyocyte-like cells	[30]
miRNA-29c	PTEN	PTEN/Akt/mTOR signaling pathway	Inhibition of excessive autophagy in cardiomyocytes	[31]
miR-126-3p	TSC1	TSC1/mTORC1 /HIF-1α /VEGFA	Promoted HUVEC proliferation, angiogenesis and migration	[127]
miR-543	COL4A1		Promotes endothelial cell angiogenesis	[128]
miR-494-3p			Promoting neovascularization after MI	[129]
miR-486-5p	MMP19	MMP19/VEGFA cleavage signaling	Increased angiogenic activity of endothelial cells	[130]
miR-132-3p	THBS1		Promoting angiogenesis after MI	[131]
miR-106a	ATG7		Inhibition of endothelial cell proliferation, autophagy	[32]
miR-409-3p	DNAJB9	MAPK	Reduced EC proliferation and migration	[33]
miR-873-5p	Cab39	Cab39/AMPK signaling pathway	Inhibition of MSC autophagy leads to MSC cell senescence	[34]
miR-155-5p	Cab39	Cab39/AMPK signaling pathway	Inhibition of MSC Aging	[35]
miR-1246	ELF5	ELF5/CD31	Promoting phenotypic transition of fibroblasts to ECs, angiogenesis and proliferation in HCF	[36]
miR-1290	SP1	SP1/CD31	Enhanced angiogenesis	[36]
miR-214-3p	PTEN	p-AKT signaling pathway	Enhanced endothelial cell migration and reduced cardiomyocyte apoptosis	[37]
miRNA-205			Promotes endothelial cell proliferation and angiogenesis	[38]
miRNA-24	eNOs		Increased angiogenesis and induced fibroblast apoptosis	[39]
miR-218-5p or miR-363-3p	p53 and JMY	p53/JMY	Promote the transformation of mesenchymal endothelial cells and inhibit myocardial fibrosis	[42]
miR-150	Sprr1a		Improvement of myocardial fibrosis after MI	[43]
miR-590-3p	ZEB1	ZEB1-Col1A1/Col3A1	Inhibition of cardiac fibroblast proliferation, differentiation, migration and collagen synthesis	[44]
miR-21	Jagged1	TGF-β1/ miR-21/ Jagged1	Promotes conversion of cardiac fibroblasts to myofibroblasts cells and myocardial fibrosis	[45]
miR-133a-3p	LTBP1 and PPP2CA； Ash1l	TGF-β pathway	Inhibition of excessive replacement fibrosis and improvement of cardiac function after MI; inhibition of macrophage M1 polarization and attenuation of cardiac inflammation	[46, 47]

Among the first identified miRNAs are miR-1, miR-133, and miR-499. In mouse teratoma-derived P19 cells - a well-established in vitro model for cardiomyocyte differentiation - miR-1 overexpression suppresses cardiac lineage commitment through dual mechanisms: enhancing cellular proliferation via upregulation of Hand2 expression, and inhibiting apoptosis by blocking caspase-3 cleavage [22]. MiR-133 can promote cardiac reprogramming through direct inhibition of Snai1 and silencing of fibroblast features to promote cardiac reprogramming [23]. Thus, miR-1 and miR-133 exert opposing regulatory effects on non-cardiomyocyte-to-cardiomyocyte transdifferentiation. Furthermore, these miRNAs demonstrate antagonistic roles in modulating post-ischemic cardiomyocyte apoptosis: elevated miR-1 and/or reduced miR-133 levels promote apoptotic cell death, whereas the inverse expression pattern enhances cell survival. This bidirectional regulation likely occurs through miRNA-mediated post-transcriptional silencing - miR-1 targets heat shock [24]. Mir-499 inhibits cardiomyocyte apoptosis by suppressing the dephosphorylation of dynamic protein-1 (Drp1) mediated by calmodulin phosphatase [25]. MiR-1 and -499 inhibit the proliferation of cardiomyocyte progenitor cells (CMPCs) and enhance the differentiation to cardiomyocytes by decreasing the levels of histone deacetylase 4 and Sox6 protein [26].

Recent advances have led to the identification of numerous miRNA that participate in post-MI cardiomyocyte regulation. Certain miRNAs suppress autophagy while promoting cardiomyocyte apoptosis through multi-pathway targeting of autophagy- and apoptosis-related genes. Conversely, other miRNAs exhibit cardioprotective effects by attenuating apoptotic pathways in cardiomyocytes. Notably, miR-181a-mediated downregulation of hexokinase 2 (HK2) led to increased mitochondrial outer membrane permeability, which resulted in apoptosis of cardiomyocytes after myocardial infarction [27], however, miR-181a directly targeted Adamts1 to regulate the level of lipid transporter protein-2 (Ngal), which improved cardiac function and inactivated the Aldo-MR pathway, proving miR-181a has a cardioprotective effect [28]; it can also play an anti-apoptotic role by targeting PDCD4 to regulate the recruitment of BID to mitochondria [29].This suggests that a miRNA may exert opposite effects through different target genes or pathways, suggesting the limitations of therapy at this target. In addition, miRNA-21 can promote the differentiation of bone marrow mesenchymal stem cells (BMSCs) into cardiomyocytes through the Ajuba/ISL1 signaling pathway [30].miRNA-29c inhibits excessive autophagy in cardiomyocytes through the PTEN/Akt/mTOR axis pathway, thereby attenuating cardiac ischemia/reperfusion injury [31].

### Neovascularization

Functional cardiac tissue regeneration necessitates sufficient vascular perfusion to meet the high metabolic demands of cardiomyocytes. Consequently, post- MI neovascularization plays a pivotal role in this reparative process. Emerging evidence indicates that multiple miRNAs modulate angiogenesis through diverse mechanisms, principally by regulating EC proliferation and migration, as well as enhancing stem cell-mediated protection and tissue repair (Table 2).

Some miRNAs promote endothelial cell proliferation by inhibiting target genes and up-regulating pathways such as vascular endothelial growth factor, thus promoting neovascularization after MI; some miRNAs exert inhibitory effects, such as miR-106a attenuates venous endothelial cell proliferation, cell cycle progression, autophagy, and angiogenesis by targeting autophagy-related gene (ATG7) [32], miR-409-3p reduces EC proliferation and migration and inhibits neovascularization and tissue repair by targeting DNAJ homologous subfamily B member 9 (DNAJB9) [33]. In addition, regulation of the transformation of MSCs or fibroblasts to endothelial cells is also a function of miRNAs. For example, miR-873-5p and miR-155-5p inhibit the mitochondrial autophagy pathway in mesenchymal stem cells (MSCs) through the AMPK signaling pathway, and lead to senescence of MSCs by inhibiting the expression of Cab39, which inhibits the vasculature after AMI Generation process [34, 35] miR-1246 and miR-1290 induce upregulation of ELF5 and SP1 by binding to their respective gene promoters. ELF5 and SP1 bind to the CD31 promoter, leading to upregulation of CD31 in human cardiac fibroblasts (HCF) and promoting phenotypic transition from fibroblasts to endothelial cells, angiogenesis, and proliferation in HCF [36]. In fact, cardiomyocyte repair and neovascularization are two closely linked processes, and the same miRNA can act on both cardiomyocytes and neovasculature, but the final biological effects may be the same or opposite. For example, miR-214-3p targeting PTEN can enhance endothelial cell migration by activating the p-AKT pathway, thereby accelerating angiogenesis in hypoxic necrotic areas and reducing myocardial cell apoptosis, thereby reducing infarct size and restoring myocardial function [37]; Adipose tissue-derived mesenchymal stem cells (ADSC)-Exos can reduce cardiomyocyte apoptosis and simultaneously increase angiogenesis through miR-205. Apoptosis while increasing angiogenesis [38]. However, local adenovirus mediated miR-24 targeted administration increases peri infarct angiogenesis and cardiac blood supply, reduces necrotic areas, promotes fibroblast apoptosis, and improves overall cardiac function [39]; However, miR-24 exhibited the opposite effect in endothelial cells by targeting transcription factor GATA2 and the p21-activated kinase PAK4 thereby inducing apoptosis in ECs [40]. This shows that miRNA-24 has different physiological effects on endothelial cells, cardiomyocytes and fibroblasts, so the treatment of MI through miRNA-24 inhibition should be targeted to fibroblasts.

### Inflammatory response

Myocardial infarction triggers a robust inflammatory response, which serves as a crucial compensatory mechanism following ischemic injury but may paradoxically contribute to adverse outcomes such as heart failure when dysregulated. Post-infarction, Toll-like receptor (TLR) signaling activation rapidly engages innate immune pathways through upregulated cytokine and chemokine production. Leukocytes infiltrate the infarcted myocardium, where they phagocytose cellular debris while dynamically modulating their own phenotypic and functional states. This inflammatory cascade subsequently recruits and activates mesenchymal repair cells - predominantly myofibroblasts and vascular smooth muscle cells - that secrete extracellular matrix components to preserve left ventricular architecture. However, excessive matrix deposition ultimately results in cross-linked collagen scar formation, compromising cardiac function [41]. Therefore, timely suppression of inflammatory reactions, especially myocardial fibrosis, is crucial for the recovery of MI. Some miRNAs are involved in this process (Table 2), for example, exo-miR-218-5p or exo-miR-363-3p up-regulate cardiac fibroblast p53 and down-regulate JMY expression, which promotes mesenchymal-endothelial transition to inhibit myocardial fibrosis [42]. MiR-150 directly targets the pro-apoptotic small proline-rich protein 1a (Sprr1a) in cardiomyocytes) in cardiomyocytes, thereby ameliorating myocardial fibrosis after MI [43]. MiR-590-3p inhibits the proliferation, differentiation, migration, and collagen synthesis of cardiac fibroblasts by suppressing the expression of ZEB1 [44]. MiR-21 promotes the transformation of cardiac fibroblasts into myofibroblasts and myocardial fibrosis by targeting JAGGED1 [45]. Serum exosomes (IPC-exo) directly target LTBP1 and PPP2CA by transfection of miR-133a-3p to inhibit excessive replacement fibrosis and improve cardiac function after MI, and the mechanism is related to the indirect regulation of the TGF-β pathway [46]; Cardiac tissue-derived EVs (ncEVs) inhibit LPS-induced macrophage M1 polarization, attenuate cardiac inflammation and improve cardiac function, while upregulating their phagocytosis via the regulation of Ash1l pathway by miR-133a-3p [47].

## miRNA and Cerebral Infarction

Cerebral infarction, clinically termed ischemic stroke, manifests as a spectrum of neurological symptoms resulting from disrupted cerebral blood supply. This condition arises from diverse cerebrovascular events including cardiac embolism, microvascular occlusion, and atherosclerotic thrombosis [48]. Post-ischemic cerebral hypoxia triggers a cascade of pathological responses - including oxidative stress, inflammatory activation, and cytotoxic edema - culminating in selective neuronal death within the ischemic penumbra [49, 50]. Distinct from myocardial infarction, neuroinflammation constitutes a fundamental pathophysiological component of cerebral infarction progression. Following ischemic stroke, cerebral hypoxia induces both necrotic and reactive oxygen species (ROS)-mediated apoptotic neuronal death, initiating a regulated inflammatory cascade characterized by: chemokine (CCL2, CXCL10) and cytokine release; resident microglial proliferation/activation; and peripheral leukocyte recruitment (monocyte-derived macrophages, neutrophils, and lymphocytes) [51].

The repair of neurovascular units after cerebral infarction is mainly through reducing neuronal apoptosis, inhibiting immune-inflammatory response, promoting neovascularization and thus repairing the blood-brain barrier (BBB) (in addition, the glial scar produced after cerebral infarction should be degraded), thus promoting neurovascular regeneration and alleviating cerebral edema and inflammation. Many miRNAs and their target genes participate in multiple pathophysiological processes related to cerebral infarction through different signal transduction pathways.

### Apoptosis and the inflammatory response

Inflammation is influenced by signaling pathways associated with multiple factors, including mitogen-activated protein kinase (MAPK), nuclear transcription factor kappa B (NF-κB), and phosphatidylinositol 3-kinase/protein kinase B (PI3K/AKT). The activation of the NF-κB pathway is closely related to the development of neuroinflammation [52]. Ischemic reperfusion (I/R) lesions can activate the NF-κB pathway, further promoting the transcription of pro-inflammatory cytokine target genes such as IL-1β, TNF-α, IL-6, and inducing inflammatory responses [53], and many miRNAs have been found to play either positive or negative roles in this neural pathway (Table 3).

**Table 3:** miRNA and cerebral infarction.

miRNA	Target gene	Signaling pathway	Corresponds	Reference
miR-155	MafB	NF-κB pathway	Promotes inflammatory response and apoptosis	[132]
miR-155-5p	DUSP14 (also known as MKP6)	NF-κB	Promotes cellular pyroptosis and inflammatory responses	[133]
miR-100-5p	TLR7	NF-κB	Promotion of neuronal apoptosis and activation of the innate immune response	[134]
miR-181	BCL2 and XIAP	NF-κB	Induction of an inflammatory response	[135]
miR-19a/b-3p	SIRT1; IGFBP3	NF-κB	Promoting inflammatory responses	[136]
miR-217	MEF2D	NF-κB; MEF2D /ND6	Enhances inflammatory response and promotes apoptosis	[137]
miR-449c-5p	STAT6	NF-κB	Activation of microglia M1 phenotype activity enhances inflammatory response	[138]
miR-3613-3p	RC3H1	NF-κB	Promoting microglia M1 polarization and thus neuronal apoptosis	[139]
miR-203a-3p/153-3p	SRC	NF-κB; MAPK signaling pathway	Reduced neuronal apoptosis and inflammatory response	[140]
miR-203	MyD88	NF-κB	reduce inflammation	[141]
miR-203-3p	Pde4d	NF-κB	Inhibition of apoptosis, inflammation and oxidative stress	[142]
miR-194-5p	GMFB	NF-κB	Promotes cell proliferation and attenuates inflammatory responses	[143]
miR-345-3p	TRAF6	TAK1/p38/NF-κB pathway	Prevention of apoptosis and inflammation	[144]
miR-26a	TREM1	TLR4 / MyD88 / NF-κB pathway	Mitigation of microglia apoptosis and reduction of inflammation	[145]
miR-378a-5p	NLRP3	NF-κB	Inhibits cellular pyroptosis, attenuates neuronal cell damage, and suppresses inflammatory responses	[146]
miR-124-3p	TRAF6	NF-κB	Reduces neuronal cell damage and inflammatory response	[147]
miR-124-5p	CYBB	NF-κB	Ditto	[148]
miR-5787	TLR4	NF-κB	Inhibition of macrophage proliferation and migration migration and inflammatory response	[149]
miR-221		NF-κB	Inhibition of pro-inflammatory responses	[150]
miR-98			Reduced monocyte infiltration and lowered the proportion of M1 microglia in the affected area, thereby protecting the BBB and attenuating the inflammatory response	[151]
miR-98	CCL2, CCL3		Protection of BBB from monocyte infiltration and prevention of M1 microglia activation	[151]
miR-449c-5p	SIRT1	SIRT1/FoxO1 pathway	Promotes microglia activation and increases BBB permeability	[152]
miR-425-5p	SIRT1	NF-κB	Promoting microglia inflammatory response and BMEC injury	[153]
miR-15a/16-1	Claudin-5		Increased peripheral immune cell infiltration, exacerbating BBB leakage	[154]
miR-34a	CYC		Increased disruption of cerebrovascular endothelial cell tight junctions	[155]
miR-29c-5p	LRP6	LRP6/TJP	Increased BMEC permeability and TJP disruption while enhancing inflammation	[156]
miR-29a-3p	SEMA3A	CircSEC11A/ miR-29a-3p/SEMA3A	Promotes BMEC oxidative stress and apoptosis and inhibits cell proliferation	[157]
miR-424-5p	FGF2	FGF2/STAT3 pathway	Promotes BMEC cell injury and exacerbates BBB permeability	[158]
miR-33a-5p	XBP1s	DANCR/miR-33a-5p/XBP1s	inhibited proliferation, migration, and angiogenesis in BMESC	[159]
miR-671-5p	MMP-9	NF-κB	Reduces TJP degradation and decreases BBB permeability	[160]
miR-23a-5p	TNF	NF-κB	Increased TJP expression and repair of the BBB barrier	[161]
miR-92b	NOX4	Foxo1/miR-92b/ NOX4	Reducing BBB damage	[162]
miR-29b	C1QTNF6	NF-κB	Reducing BBB destruction and brain damage	[163]
miR-539	SNAI2	SNAI2/MMP9	Inhibits vascular endothelial cell proliferation and decreases BBB permeability	[164]
miR-150-5p	MLLT1	circVRK1/ miR-150-5p/ MLLT1	Reducing BMEC damage	[165]
miR-429	SNAI2	SNAI2/GSK-3β/β-catenin pathway	Inhibition of angiogenesis in BMEC	[166]
miR-181b and miR-486	PTEN	PTEN /Akt pathway	Promotes proliferation of endothelial cells and thus angiogenesis	[167, 168]
miR-185-5p	IGFBP-2	ADAMTS9-AS2/ miR-185-5p/ IGFBP-2	Promotes angiogenesis	[169]
miR-21-5p	RECK	VEGF/VEGFR	Promote HUVEC proliferation, migration and angiogenesis	[170]
miR-142-5p	ADAMTS1	VEGF/PI3K/AKT	angiogenesis	[76]
miR-340-5p	CD147		Promotes angiogenesis	[171]
miR-210			Inhibition of apoptosis promotes cell proliferation and migration in HUVESCs, thereby promoting neovascularization	[172, 173]
miR-126	PIK3R2	PI3K/AKT	Promotes angiogenesis	[174]
miR-199a-5p	VEGF and BDNF		Promotes angiogenesis and neuronal regeneration	[175]
miR-214-3p	FKBP5	circ_0007865/miR-214-3p/FKBP5	Promote growth, proliferation and migration of HBMEC and inhibit apoptosis	[176]
miR-6867-5p	TWIST1		Promotes angiogenesis and decreases BBB permeability	[177]
miR-7	KLF4/VEGF and ANG-2		Inhibition of angiogenesis	[77]
miR-203	SLUG		inhibited the proliferation, invasion and migration of HUVEC	[178]

Certain microRNAs (miRNAs) promote apoptotic and inflammatory processes through target gene suppression and NF-κB pathway activation, which accelerates pro-inflammatory cytokine release and enhances leukocyte infiltration, thereby exacerbating neuroinflammation. These miRNAs, predominantly expressed in microglia, not only activate NF-κB signaling but also facilitate apoptosis by polarizing microglia toward the pro-inflammatory M1 phenotype while suppressing the anti-inflammatory M2 phenotype. Conversely, other miRNAs exert neuroprotective effects following cerebral infarction by: inhibiting inflammatory cytokine and chemokine release to attenuate apoptosis and inflammation; and suppressing the p38 MAPK/MMP-9 pathway to mitigate acute ischemic brain injury.

### Blood-brain barrier and neovascularization

BBB disruption following cerebral infarction primarily results from neuroinflammatory processes, which may precipitate hemorrhagic transformation in ischemic stroke due to degradation of tight junction proteins (TJPs; including claudin, occludin, and ZO-1) between brain microvascular endothelial cells (BMECs). During the acute phase, inflammatory mediators derived from activated brain cells and infiltrating leukocytes exacerbate endothelial injury. Notably, certain upregulated miRNAs amplify BBB damage by both potentiating inflammatory cascades and promoting TJP proteolysis in cerebral endothelial cells. Some miRNAs, on the other hand, are able to promote BBB repair and thus exert a protective effect. By targeting genes or acting on the NF-κB pathway, they attenuate the degradation or increase the expression of TJP in cerebrovascular endothelial endothelial cells, thereby reducing BBB permeability. For example, miR-671-5p targets NF-κB and attenuates TJP degradation by decreasing MMP-9 expression, thereby reducing BBB permeability. In addition, many miRNAs have an active effect on the process of neovascularization after cerebral infarction, and the main mechanism is to enhance the expression of vascular endothelial growth factor (VEGF) and inhibit the expression of endothelial repressor by acting on the target genes, thus promoting the growth and reproduction of endothelial cells, which all negatively affect angiogenesis (Table 3).

## Therapeutic approaches for targeting miRNAs

Given the role of miRNAs in the developmental process of atherosclerosis, heart infarction, and cerebral infarction, many ways exert therapeutic effects through the involvement of miRNAs, including chemopharmacological therapies, synthetic specific vectors loaded with or recognizing/targeting miRNAs (e.g., nanoparticles, circulating microparticles, and engineered exosomes), and other regulatory non-coding RNAs (e.g., lncRNAs, circRNAs) regulation, etc.

### Treatment of atherosclerosis

Many chemicals are involved in the treatment of miRNA and atherosclerosis, which involves direct activation or inhibition of miRNA expression in target cells by chemicals, modulation of miRNA delivery by exosomes from different sources, and indirect regulation of miRNAs by modulating lncRNAs or circRNAs (Table 4). They directly or indirectly up-regulate or down-regulate miRNAs to ameliorate atherosclerosis and enhance plaque stability. Among them, Salvianolic acid, the active constituent of Salvia miltiorrhiza, demonstrates particular efficacy by differentially regulating gene expression in macrophages and endothelial cells. Specifically, it upregulates let-7g in macrophages while downregulating miR-338-3p in endothelial cells, resulting in inhibition of foam cell formation, protection against endothelial cell apoptosis, and endothelial cell-derived extracellular vesicle-mediated transfer of miR-204-5p to smooth muscle cells (SMCs), which activates endothelial cell autophagy and prevents apoptotic cell death.Anthocyanin-3-O-glucoside miR-204-5p can be simultaneously inhibited in endothelial cells, indicating that the same miRNA may be chemically regulated differently; miR-135a-5p is a direct target of circ_0000231 and also targets CLIC4, astragaloside IV attenuates atherosclerosis by inhibiting circ_0000231; Salvianolic acid B may inhibit the expression of MMP9 and mmp12 in macrophages by upregulating mir-34a-5p, reducing inflammatory cell infiltration and Th1 cell response, and maintaining atherosclerotic plaque stabilization; however, it should be noted that downregulation of miR-34a-5p enhances protection against infarction, suggesting that the medication should be used with attention to the course of atherosclerosis development.

**Table 4:** Drug treatment of atherosclerosis.

Veterinary drug	miRNA	Target gene/signaling pathway	Corresponds	Reference
Atorvastatin	miR-26a-5p	PTEN	Enhancement of HUVEC viability, inhibition of apoptosis and migration	[179]
Tetrandrine	miR-34a	Wnt5a/Ror2/ABCA1/NF-kB pathway	Promotes cholesterol efflux and inhibits expression of inflammatory factors	[180]
Paeonol	miR let-7g	HMGA2/CEBPβ pathway	Inhibits foam cell formation and adipocyte differentiation	[181]
Paeonol	miR-338-3p	TET2	Enhancement of VEC viability and inhibition of apoptosis	[182]
Rapamycin	miR-204-5p		Prevention of EC apoptosis and attenuation of SMC calcification	[183]
Rapamycin	miR-155		Increased autophagic activity in carotid plaques	[184]
Cyanidin-3-O-glucoside (C3G)	miR-204-5p	SIRT1	Inhibition of HUVEC apoptosis, Inhibition of inflammation	[185]
Curcumin	miR-125a-5p	SIRT6/ ABCA1	Promote cholesterol transport and inhibit the formation of foam cells	[186]
Curcumin	miR-124	lncRNA MIAT/miR-124	Attenuates ox-LDL-induced cellular inflammation	[187]
Yi Mai granule	miR-125a-5p	Mfn2-Parkin access road	Enhanced mitochondrial autophagy in endothelial cells	[88]
Hydroxysafflor yellow A	miR-429	SLCA7A11	Inhibition of iron death in HUVEC	[188]
Aucubin	miR-181a-5p	STING	Inhibition of NF-κB pathway inflammation	[189]
astragaloside IV	miR-17-5p	PCSK9/VLDLR signaling pathway	Inhibits vascular inflammation, thereby attenuating endothelial cell damage	[190]
astragaloside IV	miR-135a-5p	circ_0000231/miR-135a-5p/ CLIC4	Inhibition of HUVEC cell damage	[191]
Theaflavin	miR-24	Nrf2/HO-1 signaling pathway	Protection of HUVEC cells from oxidative damage	[192]
Notoginsenoside R1 (NGR1)	miR-221-3p	XIST/miR-221-3p/TRAF6 Axis	Promotes HUVEC proliferation and inhibits apoptosis death, inflammation, and oxidative stress	[193]
Salvianolic acid B	miR-34a-5p	MMP9 and MMP12	Reduction of inflammatory cell infiltration and maintenance of atherosclerotic plaque stabilization	[194]
dihydromyricetin	miR-21	DDAH1/ ADMA	Increased endothelial cell NO synthase (eNOS) phosphorylation and NO production	[195]
L-Arginine	miR-221	eNOS	Inhibits oxidized LDL white-induced apoptosis in endothelial cells	[196]
bosentan	miR-21	PDCD4	Prevents endothelial cell death	[197]
Tanshinone IIA	miR-130b	WNT5A	Inhibits foam cell production and inflammation	[198]
Tanshinone IIA	miR-375	KLF4	Enhancement of macrophage autophagy as well as M2 polarization	[199]
Nobiletin	miR-590	LPL	Reduces lipid accumulation and inflammation	[200]
Ojeoksan	miRNA-10a、-126-3p	eNOS and MMP	Increased endothelial cell NO synthase (eNOS) phosphorylation and NO production	[201]
ursolic acid	miRNA-21	PTEN/PI3K	Inhibition of smooth muscle cell proliferation	[202]
Diosgenin	miR-19b	ABCA1	Enhanced macrophage cholesterol efflux	[203]
coenzyme Q10	miR-378	ATP-binding cassette transporter protein G1	Enhanced macrophage cholesterol efflux	[204]
Tetramethylpyrazine and paeoniflorin combination therapy (TMP-PF)	miR-1268b	circSCRG1/miR-1268b/NR4A1	Inhibits HUVEC angiogenesis and increases plaque stability	[205]

An increasing number of studies have been conducted by designing nanoparticles with the ability to load miRNAs (or other substances) and target specific cells. By using click chemistry technology to modify the hyaluronic acid (HA) on the surface of LSS EV, it can specifically bind to the CD44 receptor on the surface of pro-inflammatory macrophages in plaques. As a result,mir-34c-5p in EVs targets the tgf- β -smad3 pathway and promotes the repolarization (reprogramming to M2) of M1 phenotype macrophages [54]. Ultrasound-targeted microvesicle disruption (UTMD) delivery of miR-145a-5p promotes a contractile phenotype in VSMCs [55]. SPION capsule miR-146a is a spherical nucleic acid nanostructure, which can independently enter macrophages and ECs, regulate the NF - κ B pathway and treat atherosclerosis. miR-146a has dual functions of targeting and gene regulation [56]. Using pH low insertion peptide (pHLIP) construct as a vector, miR-33 antisense oligonucleotide was delivered into macrophages, which binds to miR-33 and thus up-regulates the expression of fibrogenic genes, Timp3, and MMP12, and is applied to treat advanced atherosclerosis [57]. Exosome engineered IL-10 mRNA has miR-155 recognition site, when miR-155 is activated, the exosome is delivered to macrophages and acts as an anti-inflammatory [58]. Inhibition of miR-126 by nanoparticles containing miRNA switches inhibits smooth muscle cell division and migration and protects endothelial cells [59]. Co-culture of advanced endothelial progenitor cells (EPC) and circulating particles (MP) improves EPC function through miR-10a, miR-21, miR-126, miR-146a, miR-223 metastasis and IGF-1 expression activation [60]. MiRNAs also play a role in preventing non target cell damage. Delivery of EV based magnetic iBax mRNA and BAX activator BTSA1 effectively promotes the death of receptor aging cells in plaques, while miR-122 overexpressed in liver cells targets BAX mRNA, thereby protecting the liver from potential damage caused by BAX mRNA [61].

### Treatment of heart attacks

As previously discussed, numerous pharmacological interventions delay atherosclerotic plaque formation and progression, while preventing plaque rupture - a critical event leading to vascular occlusion and subsequent infarction - represents another key therapeutic strategy. Many drug therapies exert therapeutic effects on heart attack by directly affecting miRNAs or indirectly regulating miRNAs through lncRNAs and circRNAs (Table 5). Exosomes derived from serum, cardiac tissue, adipose tissue, macrophages, MSCs, and other sources play a role in repairing post-infarction damage by transfecting miRNAs, and therefore therapeutically significant exosomes can be targeted to target cells by means of physical techniques or chemical agents. For example, miR-132-3p was enriched in M2 macrophage-derived exosomes (M2-exos), which were translocated into endothelial cells to promote post-MI angiogenesis by directly targeting THBS1. Pretreatment of exosomes from MSCs with vericiguat (MSC(VER)-Exo), applied to cardiac fibroblasts, inhibited fibroblast proliferation, migration, and pro-fibroblast gene expression, thereby attenuating cardiac fibrosis, in which miR-1180-3p, which targets ETS1, plays an effective role in anti-fibroblasts [62]. MiR-125b is a cardiac infarction miR-125b is an important miRNA molecule in infarction. Targeted delivery of MSC membrane ligands and miR-125b to the inflamed region of AMI using ultrasound-targeted microbubble disruption (UTMD) prevents cardiomyocyte death and inhibits the growth of fibroblasts [63]. In addition, precise cardiac-specific genome editing can be performed with combining extracellular vesicles (EV) with cardiac-targeting peptides (T). RNP complexes of single guide RNA targeting miR-34a were loaded into EV, which inhibited miR-34a expression and attenuated apoptosis of cardiomyocytes [64]. C166-derived EV was also an effective deliverer of miRNA combinations in vitro and in vivo, delivering miR-148a-3p to fibroblasts and induces reprogramming of fibroblasts into cardiac muscle cells by targeting Mdfic [65]. Intramyocardial injection of cardiopulmonary progenitor (CPP) cells improves cardiac function by promoting cardiomyocyte proliferation and vascularization via exosomes of CPPs (CPPs-Exo). It has been shown that high expression of miR-27b-3p and its target gene, Sik1, in CPPs-Exo affects the transcriptional activity of CREB1 [66].

**Table 5:** Drugs for the treatment of myocardial infarction.

Veterinary drug	miRNA	Target gene/signaling pathway	Corresponds	Reference
Carvedilol	miR-125b-5p	circ_NFIX/ miR-125b-5p/ TLR4	Protection of H9c2 cells from H2O2-induced cellular dysfunction	[206]
quercetin	miR-221	circPAN3/ miR-221/PTEN	Reduction of cardiomyocyte apoptosis	[207]
Qili Qiangxin Capsule (QLQX)	miR133a	GRP78, IRE1, ATF6 and XBP1	Reduced cardiomyocyte apoptosis	[208]
Astragaloside IV	miR-411	HIF-1α	Improves heart function and promotes neovascularization	[209]
atorvastatin (ATV)	miR-139-3p	Stat1 pathway	Promoting macrophage polarization and cardiac repair	[210]
ginsenoside Rg3	miR-128-3p	MDM4	Inhibited cardiomyocyte apoptosis and oxidative stress	[211]
Melatonin	miR-200b-3p	HMGB1	Promotes H9c2 cell proliferation and inhibit apoptosis in ischemic environment	[212]
EEpigallocatechin gallate	miR-450b-5p	ACSL4	Mitigating AMI-induced iron death	[213]
IFN-γ-Exo	miR-21	BTG2	Promotes angiogenesis and reduces apoptosis	[214]
take care of the heart’s health (TCM)	miR-146a-5p	IRAK1 / NF-κB p65	Reduces cardiomyocyte apoptosis and inflammation	[215]
Tanshinone IIA (Tan IIA)	miR-499-5p	PTEN	Promotes proliferation and migration of HUVEC and angiogenesis	[216]
propofol	miR-206	MALAT1/miR-206/ATG3	Protects cardiomyocytes from I/R damage	[217]
Danhong Injection (DHI)	miR-125b	p53 myocardial apoptosis pathway	Attenuating apoptosis in the heart after MI	[218]
Photobiomodulation (PBM)	miR-136-5p	Ino80/ miR-136-5p	Promote cardiomyocyte proliferation	[219]

### Treatment of cerebral infarction

Extracellular vesicles containing superparamagnetic iron oxide nanoparticles (SPION-EX) can effectively penetrate the BBB and enter neurons in brain tissue, improving mitochondrial function of post-stroke neurons through the miR-1228-5 p/TRAF 6/NOX 1 signaling pathway [67]. MiR-21-5p is transferred from ADSC exosomes to microglia to promote M2 polarization and alleviate inflammation through the PIK3R1/PI3K/AKT cell pathway [68]. MSC-derived exosomes (Hypo-Exo) cultured under hypoxic conditions containing miR-214-3p promote angiogenesis through the PTEN/Akt pathway [69]. A probe with aggregation-induced emission (AIE) properties (i.e., TTCP) that efficiently labels EC-EV, which carries miRNA-155-5p is taken up by astrocytes and promotes neurological restoration by targeting the c-Fos/AP-1 pathway [70]. By pre-treating astrocytes with berberine, it is possible to induce the release of extracellular vesicles (BBR exos) carrying miR-182-5p from damaged neurons, and Rac 1 inhibits neuroinflammation and improves expression of brain injury after cerebral [71]. The delivery of miR-124 through Ca-MOF nano delivery system can significantly promote the internalization of miR-124 in neural stem cells (NSCs) and promote the differentiation of NSCs into mature neurons [72]. Circ-Rps5 modified ADSC exosomes improve cerebral infarction by alleviating neuronal damage and converting microglia from M1 phenotype to M2 phenotype in the hippocampus, which mechanism is to downregulate miR-124-3p, thereby upregulating SIRT7 [73]. Circ-Rps5 modified ADSC exosomes improve cerebral infarction by alleviating neuronal damage and converting microglia from M1 phenotype to M2 phenotype in the hippocampus. The mechanism is to downregulate miR-124-3p, thereby upregulating SIRT7 [74]. Nanoparticles coated with peptide nucleic acid (PNA) or phosphorothioate (PS) anti-miRs-141-3p probes reduce the production of the pro-inflammatory cytokine TNF-α [75].

Some chemical drugs are also involved in the treatment of cerebral infarction through miRNAs (Table 6). In addition, electroacupuncture has been found to be a useful therapy for cerebral infarction, and several studies have elucidated its mechanism of treating diseases by regulating miRNA. For example, electroacupuncture therapy upregulated the expression of miR-142-5p and inhibited its target gene ADAMTS1, thereby promoting the VEGF/PI3K/AKT/eNOS pathway, which led to a reduction in the infarct area [76]. Reduced miR-7 expression after electroacupuncture deregulated the downstream target genes KLF4/VEGF and ANG-2, thereby promoting post-infarction angiogenesis [77]. Acupuncture treatment upregulated the expression of miR-34c-5p and enhanced cellular autophagy, which was beneficial to cerebral infarction treatment, and its combination with the autophagy agonist RAPA enhanced the therapeutic effect [78]. Pretreatment of MSC with lithium altered the EV secretion pattern, and Li-EV inhibited the NF-κB signaling pathway by targeting TLR4 through the delivery of miR-1906, resulting in a reduction of inflammation levels [79]. Nespas expression was significantly elevated after transcranial focused ultrasound stimulation (tFUS) treatment, which down-regulated miR-383-3p expression, deregulated its inhibition of the target gene SHP2, and inhibited the production of pro-inflammatory cytokines in microglia [80].

**Table 6:** Drug treatment of cerebral infarcts.

Veterinary drug	MiRNA	Target gene/signaling pathway	Corresponds	Reference
Tanshinone IIA	miR-124-5p	FoxO1	Inhibition of inflammatory response and neuronal apoptosis	[220]
berberine (medicine)	miR-377-3p	METTL3/ NEAT1/miR-377-3p	Neuroprotective effects	[221]
Gualou Guizhi decoction	miRNA210	HIF/VEGF	Enhanced angiogenesis	[222]
dexmedetomidine	miR-665	ROCK2 and NF-κB p65	Reduces inflammation and apoptosis	[223]
Ginseng Yangrong decoction (GSYRD)	miRNA-210	HIF/VEGF/Notch signaling pathway	Promotion of neovascularization and cerebral protection after ischemic brain injury	[224]
Gastrodin (GAS)	miR-22-3p	lncRNA NEAT1/miR-22-3p	Attenuates I/R-induced inflammation in neuronal cells	[225]
Qingda granule (QDG)	miR-137	lncRNA GAS5/miR-137	neuroprotection	[226]

The core pathophysiological basis of myocardial infarction and cerebral infarction is the same, that is, atherothrombosis leads to acute obstruction of the main blood supply artery or its branches, which directly leads to blood flow interruption and insufficient oxygen supply. Interventions targeting these upstream Pro thrombotic and vascular disease miRNAs (such as antagonists or mimics) can theoretically reduce the risk of myocardial infarction and cerebral infarction at the same time, and play a role in preventing the occurrence of disease. After myocardial infarction and cerebral infarction, cells in the heart and brain are subjected to ischemia / reperfusion injury. At this time, broad-spectrum neuroprotective miRNA drugs that can alleviate ischemia / reperfusion injury (such as targeting inflammation, oxidative stress, cell death, repair remodeling, etc.) can be developed, so as to reduce organ damage and promote tissue repair. It should be noted that although the target pathways have similarities, the final effect still needs to be fully verified in specific cell types of their respective organs to avoid off target effects. At the same time, the location of cerebral infarction is different from that of myocardial infarction. Due to the existence of blood-brain barrier, targeted drug delivery after cerebral infarction is more difficult, which should also be noted.

## Challenges and Development Strategies

This review aims to provide new therapeutic approaches for cerebral infarcts and infarcts caused by atherosclerosis. However, we found that there are many challenges in targeting miRNAs for cerebral and cardiac infarction. MiRNAs are small non-coding RNAs with specificity, which can regulate gene expression by degrading or inhibiting the translation of multiple target mRNAs, with a low precision, and the principle of action of miRNAs in cardiac and cerebral infarction is complex and diverse, different miRNAs may exert different effects at different pathological stages. Therefore, there is a need to utilize tissue-specific promoters or enhancers to drive miRNA expression in specific tissues, or to design synthetic miRNAs to be active only in specific cell types, e.g., by binding to cell-type-specific RNA-binding proteins, to avoid potential side effects [81].

Second, the delivery system for miRNA therapy is an important challenge. Due to the molecular nature of miRNAs, their stability and targeting in vivo is limited. Researchers are exploring a variety of delivery systems, such as nanoparticles, liposomes, polymers, and other novel delivery vehicles, to improve the delivery efficiency and targeting of miRNA. For example, low molecular weight heparin-modified nanocomplexes have been used to improve miRNA delivery in infarcted regions, showing favorable therapeutic effects. Alternatively, viral vectors, such as adenovirus or lentivirus, are utilized for tissue-specific delivery, addressing both immunogenicity and toxicity. Exosomes also have potential as natural delivery vectors and can be utilized for their natural intercellular communication [82].

In addition, in terms of safety, miRNA modulation may affect multiple physiological pathways, and therefore, it is necessary to evaluate long-term safety, including potential off target effects and side effects. Rigorous in vitro and in vivo toxicity tests, including cytotoxicity, immune response, and genotoxicity, are required during experimentation and translation, as well as the use of bioinformatics tools to predict off-target effects of miRNAs and validate them in preclinical studies.

In recent studies, some new targets involved in atherosclerosis have been found. For example, Apelin/APJ mediates endoplasmic reticulum autophagy by upregulating SEC62 as an endoplasmic reticulum autophagy receptor protein, and upregulating the expression of adhesion molecules ICAM-1/VCAM-1, thereby promoting monocyte adhesion to vascular endothelium [83]; CYSLTR2 and P2RY6 are potential endogenous receptors for C16:0 ceramide induced inflammasome activation in endothelial cells and macrophages, increasing the risk of atherosclerosis [84]; Liver kinase B1 (LKB1) inhibits the phenotypic transformation of vascular smooth muscle cells by activating SIRT6, thereby delaying the onset of atherosclerosis [85]; Targeting the PGAM5-ANGPT3 signaling axis by inhibiting PGAM5 can reduce inflammation and improve macrophage lipid metabolism [86]. However, the research on the targeting effect of miRNAs on these proteins and potential therapeutic options are still blank.

In addition, in addition to the mainstream cells involved in atherosclerosis (endothelial cells, macrophages, vascular smooth muscle cells), other immune cells (such as proinflammatory T cells) may also have therapeutic targets. A study showed that PD-1 monoclonal antibody can inhibit plaque activation and proinflammatory PD-1 positive T cell function, thereby alleviating atherosclerosis, suggesting that we can also explore the targets of miRNAs in T lymphocytes [87].

In conclusion, although targeted miRNA therapies show great potential in heart and brain infarction, their clinical application still needs to overcome multiple challenges such as delivery system, specificity and safety. Future studies should continue to explore in depth the mechanism of miRNA action in cardiovascular and cerebrovascular diseases and develop more efficient and safe therapeutic strategies.

## Conclusion

This review organizes the involvement of miRNAs in atherosclerosis and the resulting heart and brain infarcts, including the three main types of cells in atherosclerosis (ECs, macrophages, and VSMCs), and the development of heart infarcts (apoptosis of cardiomyocytes, angiogenesis, etc.) versus brain infarcts (neuroinflammatory response, BBB repair, etc.) after plaque rupture. In addition, various therapeutic means for targeting miRNAs, such as drug therapy, engineered exosomes, acupuncture therapy, etc., are also highlighted and summarized. MiRNAs, as a key gene expression regulator, play an important role in the development and progression of atherosclerosis. By targeting specific miRNAs, the expression of atherosclerosis-related genes can be effectively regulated, thus achieving the purpose of treating cerebral and cardiac infarction. miRNAs’ strategy of treating cerebral and cardiac infarction by regulating atherosclerosis not only reveals the molecular mechanism of disease occurrence, but also provides new targets and ideas for clinical treatment. In the future, with the in-depth study of miRNA function and the continuous progress of technical means, targeted miRNA therapy is expected to become an important means in the field of CVD treatment, which will bring more effective therapeutic choices and better quality of life for patients.

## Availability of data and Materials

From Pubmed Data base.

## Data Availability

From Pubmed Data base.
